# Observations on the Pathology of Angina Pectoris

**Published:** 1831-07

**Authors:** Charles Mitchell

**Affiliations:** Surgeon.


### ANGINA PECTORIS.

### *Observations on the Pathology of Angina Pectoris.*

By Charles Mitchell, Surgeon.

The painful and harassing affection, which has been in most instances denominated Angina pectoris, appears to be nothing more than irritability of the thoracic nerves, analogous to that state of the mammæ described by Sir A. Cooper under the appellation Irritable, and also to that state of the joints to which the same term is assigned by Mr. Brodie. The late Dr. Gooch has described a similar state of the uterus, under the title and character of irritable, which was formerly denominated Hysteralgia: he has faithfully detailed some rare, obstinate, and valuable examples.

The technical term, therefore, of Drs. Heberden, Parry, and other distinguished authorities, does not seem to have been established by proper data or legitimate deduction; for in the instances where we have most expected organic lesion in the valvular apparatus of the heart, we have failed, after minute inspection, in discovering the least apparent vestige of disease, and have often found, upon dissection, disorganization of the valves of the heart, where no symptoms had existed, during life, which could at all indicate the existence of such lesion.

Malconformation of the valves must be looked upon as incurable, and the vague hypothesis of ascribing the phenomena of a disorder (for it appears functional) to an alteration of organised parts, has materially obstructed the relief which might have been afforded by medicines used in analogous disorders of the nervous system; the failure of which, no doubt, would have stimulated to farther research, for the remedies we are as yet acquainted with cannot be defined, with any degree of propriety and consistency, beyond the natural limits of palliatives; for their efficacy has never, as far as my experience and knowledge have extended, appertained to any degree of stability, so as to be pronounced permanent.

This disorder is productive of repeated paroxysms of suffering, with occasionally alternate degrees of relaxation: during the repose, however, the twitchings have been very readily excited, by pressure so as to produce friction against one of the ribs; at times, a tap with the fingers against the fore or lateral regions of the chest has readily excited them, in cases of greater sensibility, the friction having been only necessary during torpidity, or, as it were, dormancy of the parts: and in cases imperfectly formed, however severe and long continued, I have never, in any one instance, observed structural change from its effects; and in this, I presume, my experience is corroborated by Dr. Gooch's interesting cases of irritable uterus.

In my opinion, it would be more correct, and more explanatory of the real nature of this disorder, to denominate it Thoralgia; for how the symptoms, which are of spasmodic character, and in their nature superficial, can be attributed to the region of the heart at the commencement, I am totally unable to comprehend; but still they are, by many practitioners, constantly referred to that region, when, upon proper inquiry, they are found to originate from functional derangements of the contiguous organs, or spasmodic twitching of the sentient extremities of the thoracic nerves: for I am greatly mistaken if this disorder will not be found to have its primary seat betwixt the common integuments and ribs, ultimately extending to become an internal complication, in consequence of the minute nervous intercourse. The secondary symptoms thus developing themselves after a long and painful continuation of the primary, then the fits begin to influence the regularity of the heart's action, producing a greater degree of tightness and anxiety. This impulse is not confined to the heart's action alone, but produces more than momentary suspension of respiration, which becomes more urgent as the disorder proceeds, attended with agony and oppression; some time elapses before the respiratory action takes place naturally. The connexion of the pulmonic with the cardiac plexuses of nerves probably accounts for the participation of the lungs in the state of excitement, thereby not only inducing some degree of stagnation in the lungs, but for a time, from the state of agitation in the nervous parts, preventing the due transmission of the nervous stamina to the heart; consequently inducing great reduction and irregularity of the heart's action, if not complete syncope, or even death. At this period of the disorder, the contractility of the heart is greatly impaired; hence we have sensations of distention, anxiety, oppression, and pain, during the attacks.

Viewing it, therefore, as analogous to neuralgic disorders, let us, first, consider tic douloureux: a twitching of the ramifications is primarily induced; it extends from the filaments to the trunk of the nerve, until finally the whole face participates in the involuntary, preternatural, spasmodic, and convulsive action. In other states, the same as in thoralgia, the twigs will be found first affected, until, by relationship and sympathy, the allied parts are involved.

The erroneous supposition which attributes this disease to an ossific or calcareous deposition in the valvular apparatus of the heart, must appear clear to every impartial and unbiassed mind; for how can we reconcile ourselves to the idea of ossification as the cause of such an agonizing malady, when this change will be found existing in one out of every three who has lived three score and ten years. Even in more extensive disorganizations, such symptoms do not occur; for they are not only chronic in most instances, but permanent, especially when the coronary and semilunar valves are converted into ossific or calcareous productions: nay, even where the ossification and calcareous matter resemble, in the internal surface of the aorta, a tubular form of crystallization.

Bleeding, blistering, and other counter-irritants, have been found serviceable in the disease termed Angina pectoris. Now, how and in what way they can be remedial in ossification of the coronary valves, I am unable to determine. I shall for the present content myself with detailing two cases, together with the post-mortem examinations.

Case I. A poor, forlorn-looking being, with sadness and anxiety depicted in his worn and wretched countenance, about sixty years of age, had been subject for years to spasmodic attacks, for which he had been bled and blistered with great advantage. The paroxysms of pain and twitching were now frequently renewed: his bowels were open; his mind easy; appetite moderate. A small quantity of blood was extracted to relieve the symptoms: the blood displayed no trace of inflammmatory action. The bleeding afforded him considerable temporary relief; the twitching and sensibility did not entirely subside, but became aggravated in the course of a month. Depletion was again had recourse to, and proved ineffectual; counter-irritants likewise failed in procuring relaxation from the torture of this harassing malady; opiates, for a time, allayed the irritability, but they were productive of stupor, headach, and costiveness. Camphor and opium, applied externally in the form of liniment, effected considerable quietude of the incited parts. Blisters were had recourse to again, but failed in making the least impression on the disorder. He now relinquished the use of medicine, finding it of no avail. Three days only elapsed, when he was seized suddenly, dropped down in a state not (I believe) of complete syncope, but bordering thereon: palliatives were again resumed, and gentle exercise encouraged: he moved about slowly, in a bent posture, by the aid of a crutch, with his hand applied as a support to the chest.

After continuing the application of the liniment, the administration of Dover's powder at bedtime, and subsequently small quantities every four hours, for ten days, he again became disinclined to take more medicine; but, from the severity and sense of suffocation, and darting nature of the paroxysms, he was constrained to try the local application of the concentrated volatile alkali to the chest, by means of a piece of sponge, which produced the ordinary symptoms of local inflammation and eruption. The relief afforded was considerable; but he was, upon the return of the fits, by no means anxious again to submit to such an application.

The spasmodic twitchings now became exceedingly violent upon movement, and he was consequently obliged to confine himself constantly to bed: he was unable to lie down; he became oppressed; breathing difficult; the pain darted with rapidity to both shoulders, down the arms, attended for some time afterwards with loss of power, and extended to the extremities of the fingers of the left side; the respiration became so impeded, that great effort was necessary for its continuance. He gradually became more oppressed and exhausted, until finally he expired.

*Sectio cadaveris.* Upon examining the muscular and cellular tissue exterior to the ribs, no morbid appearance was traceable. Upon the removal of the sternum and true ribs at their cartilages, the left cavity of the thorax was found completely distended with fluid; the left lung was collapsed, so as to be very little bigger than a large clenched hand: it was otherwise unaltered in its texture and structure, excepting as far as a greater degree of density was concerned. The structure and textures of the heart were natural.

This insidious disorder was removed, at the commencement, by depletion: how far predisposition influenced its renewal, or fresh exciting causes, it would be hazardous to conjecture; but the disorder became more confirmed as depletory measures became the more necessary, and were persisted in; for they afforded considerable temporary relief, probably by removing or diminishing the congested state of the excited parts; the neurilema, I presume, participating at the commencement, or, at least, the vessels of the parts were distended so as to produce pressure against the nervous fibrillæ. Be it, however, as it may, the connexion increased, until the whole nerves of the thorax became involved, producing a complicated, tedious, and fatal disorder, which primarily impaired the vital powers of the system, by inducing debility and effusion, and by destroying the power of the absorbent vessels. The sense of suffocation was so great during the severe paroxysms, that death must have ensued if great effort had not been exerted by the patient with the voluntary muscles of respiration. Soothing means certainly were productive of most benefit in the disorder, when it acquired in a more marked degree the character of nervous; for the dissolution appears to have been hastened by the irritation produced by the local application of the concentrated volatile alkali.

Case II. An athletic man, fifty-six years of age, for the last fifteen months has been under my care, at repeated intervals, for moving and twitching pains of the side, extending backwards to the spine, forwards to the sternum, and upwards to the shoulder, induced by taking a deep inspiration, or using exertion. Depletion and purgatives produce a subsidence of the paroxysms; but in December the attacks became more irrelievable and acute, by embarrassing the breathing, (as if electric sparks were transmitted through the thorax,) and considerably impeding the circulation. Bleeding, antispasmodics, aperients, and counter-irritants, proved very advantageous, by diminishing the severity of the twitchings and oppression: they soon, however, increased. Bleeding was again resorted to, with a view of amelioration, yet the pains were now no longer fleeting and transitory, but had acquired a greater degree of permanency. The internal administration of opium proved ineffectual: the external use of the same remedy appeared to deserve more preference, for the rubbing of the Liniment. Sap. c. Opio over the fore and lateral regions of the chest tended greatly to suppress, allay, and remove the paroxysms of anxiety, dyspnœa, and spasmodic action. This system of palliation failed in the course of one week; for the irritability of the thorax had become remarkable, although the sensibility had been blunted by the opiate, the twitchings being readily induced by pressure. A large blister was applied, small doses of bluepill and Dover's powder given internally, with decided amendment for a few days; and, upon the increased recurrence of the spasms, camphor and ammonia mitigated the obscure, perplexing, and unaccountable phenomena.

The symptoms being relieved, I discontinued my attendance, but in about a month I was requested again to visit him. I found him propped up in bed by pillows; the breathing laborious, mouth wide open, pulse scarcely perceptible, and a cold clammy sweat pervaded the whole frame.

*Autopsy*. No morbid alteration could be observed in the brain, chest, or abdomen, excepting a collection of serous fluid in both cavities of the chest, not exceeding one pint: the left side contained the greater proportion.

*Lamb's Conduit street; May* 14, 1831.

